# Apoptotic extracellular vesicles: emerging biomarkers for cancer and neurodegenerative disease diagnostics

**DOI:** 10.3389/fimmu.2025.1619456

**Published:** 2025-11-21

**Authors:** Ehsan Soleymaninejadian, Marziyeh Sadeghi Dehkordi

**Affiliations:** 1 Department of Health Sciences, University of Milan, Milan, Italy; 2 Department of Biology, Faculty of Science, Shahrekord University, Shahrekord, Iran

**Keywords:** extracellular vesicles, cancer diagnostics, neurodegenerative diseases, isolation techniques, biomarkers, clinical translation, standardization, personalized medicine

## Abstract

A new subclass of extracellular vesicles formed on terminal apoptosis, apoptotic extracellular vesicles (ApoEVs), has been found to be potential markers for disease diagnosis, prognosis, and monitoring of treatment. Highlighting their role in neurological disorders and cancer as diagnostic markers, this review aims to present a new paradigm of ApoEV classification based on their biogenic process, cargo composition, and functional attributes. The heterogenous molecular content of such membrane-bound vesicles-like proteins, lipids, DNA, and RNA produces unique fingerprints reflective of the pathologic and physiological status of their parent cells. We critically examine the clinical significance, specificity, and sensitivity of current technological advancements in identifying and fractionating ApoEV through methodologies like flow cytometry, imaging processes, and molecular analysis. The prognostic and diagnostic potential of ApoEVs in cancer and neurological disease is the specific focus of this review. We address conflicting evidence, discuss controversy in the field, and contrast ApoEV-based methods with traditional biomarkers. We discuss the challenges of isolating, detecting, and validating ApoEVs and provide a standardized diagnostic procedure for clinical application. Lastly, we outline the next-generation research directions such as AI-assisted ApoEV analysis, emerging biosensor technologies, and ApoEV platforms for the specific purpose of application in personalized medicine. Finally, this thorough review critically evaluates the biological and technical hurdles which should be addressed for successful clinical translation, and the untapped potential of ApoEVs as non-invasive diagnostics.

## 1 Introduction

Extracellular vesicles (EVs) have emerged as critical mediators of intercellular communication and potential biomarkers for various diseases. EVs are present in most physiological fluids and are released by most cell types (1–7). Exosomes, microvesicles, and apoptotic extracellular vesicles (ApoEVs) belong to the heterogeneous population of EVs (8–12). **Table 1** demonstrates the varies features of different member of EVs.

**Table 1:** Members of EVs family and their characteristics.

Feature	Exosomes (Small EVs)	Microvesicles (MVs) / ectosomes	ApoEVs and ApoBDs
Biogenesis Pathway	Endosomal pathway (MVB-PM fusion).	Budding/shedding from the plasma membrane of viable cells.	Caspase-mediated fragmentation (ROCK1/PANX1 regulation).
Size Range	30 – 150 nm (Relatively Homogenous)	100 – 1000 nm (Variable)	Heterogeneous Population
Density (Sucrose Gradient)	1.10 – 1.21 g/mL (High endosomal protein content)	1.04 – 1.07 g/mL (Low density)	1.08 – 1.30 g/mL (Variable due to internal DNA/organelles)
Key Surface Markers	Tetraspanins (e.g., CD63, CD81), Alix, TSG101, Hsp70/90.	Cell-specific markers, Integrins. Phosphatidylserine (PS) externalization (inconsistent).	Phosphatidylserine is definitive, Thrombospondin, Caspase fragments.
Signature Cargo	miRNA, mRNA, endosomal proteins (Alix, TSG101), specific lipids (ceramide).	Cytosolic proteins, full-length mRNA/miRNA, specific receptors.	Nucleosomes/fragmented chromatin, caspase fragments, mitochondria.
Clinical Utility	Molecular transfer, immune modulation, general cell status indicator.	Coagulation, inflammation signaling, cell-specific communication.	Better indicator of cell death/apoptosis, monitoring therapeutic efficacy.

ApoEVs represent an underexplored yet potentially valuable source of prognostic and diagnostic data, although exosomes and microvesicles have received much attention in biomedical research.

ApoEVs are membrane-bound, EVs (1–5 um) secreted during the later phases of apoptosis. Differently from other EV subtypes, ApoEVs is enriched in nuclear material, organelles, and debris from apoptotic cell indicative of its terminal status (13). Such distinct content yields precious information on disease mechanisms by capturing the cellular state at the moment of cell death. Because of their potential applications in immune regulation, disease biomarker discovery, and therapeutics, research on ApoEVs has accelerated in recent years (14).

Despite its clinical promise for application, various practical limitations have so far discouraged the routine implementation of ApoEVs as routine biomarkers. These include the technical constraints of isolation and characterization protocols, heterogeneity of ApoEV populations, and the lack of standard operating procedures for their clinical application. Furthermore, the specificity and validity of ApoEVs as diagnostic biomarkers have been questioned by contradictory reports on the nature of their payloads and functional properties. This article aims to address these questions by presenting an in-depth review of ApoEVs as biomarkers, with a particular focus on their relevance in cancer and neurological disorders. It should be noted that we used ApoEVs term as an umbrella term for not only smaller vesicles but also the larger particles such as apoptotic Bodies (ApoBDs). To further define the diagnostic value of ApoEVs, we present a novel classification system based on their biogenesis, cargo, and functional properties. We will also compare sensitivity, specificity, and clinical utility of current detection and isolation strategies. It should be noticed that in the case of size and surface markers distinguish between ApoEVs and normal EVs is difficult (15). In contrast to previous reviews that have examined ApoEVs across a broad spectrum of diseases, we have specifically focused on cancer and neuro-degenerative disease, since they are two diseases in which ApoEVs show particularly strong potential as biomarkers. This targeted approach enables a closer examination of the specific opportunities and challenges in these areas. We discuss controversies, evaluate conflicting data, and compare ApoEV- based methods with validated biomarkers. We conclude by outlining novel research opportunities, such as AI-facilitated ApoEV analysis and emerging biosensor methodologies, and suggesting a streamlined diagnostic protocol to be applied to the clinic. The review presents an in-depth approach for maximizing ApoEV translational utility in cancer and neuro-degenerative disease diagnosis and monitoring examining both their potential and limitations as biomarkers.

## 2 ApoEV biogenesis and characteristics: a novel classification framework

### 2.1 Mechanisms of ApoEV formation

ApoEVs are distinguished from exosomes and microvesicles by their origin from distinct cellular processes specifically, ApoEVs are generated during the late stages of apoptosis as the result of a complicated process of membrane blebbing and fragmentation of cells (1, 16, 17). In general, ApoEVs are larger compared to exosomes and microvesicles, ranging from 50 nm to several micrometers in diameter (1).

The most recent studied indicate that ApoEVs production is not merely a random cell fragmentation, but it is driven by a tightly coordinated process. Specific molecular pathways, such as the activation of Rho-associated protein kinase 1 (ROCK1) and the formation of membrane protrusions known as apoptopodia. Inhibition of Pannexin 1 (PANX1) channel has also been shown to influence the release of ApoEVs by modulating this string-like membrane structure (18).

We suggest a new classification of ApoEV biogenesis pathways based on new data: Large membrane bleb formation with cellular contents, actin-myosin contraction, and ROCK1 activation characterize the Classical Membrane Blebbing Pathway (19, 20). The development of beaded apoptopodia with the shedding of smaller and more homogeneous ApoEVs containing different cargo profiles is described as the Apoptopodia-Mediated Pathway (14, 21). And, PANX1-Regulated Pathway This pathway is defined by the regulation of nuclear content incorporation into ApoEVs via PANX1 channels (22, 23).

The size range heterogeneity and cargo content variability seen between studies may be explained by this classification, which offers a more general explanation for ApoEV heterogeneity.

### 2.2 Morphological and molecular characteristics

**Figure 1:**
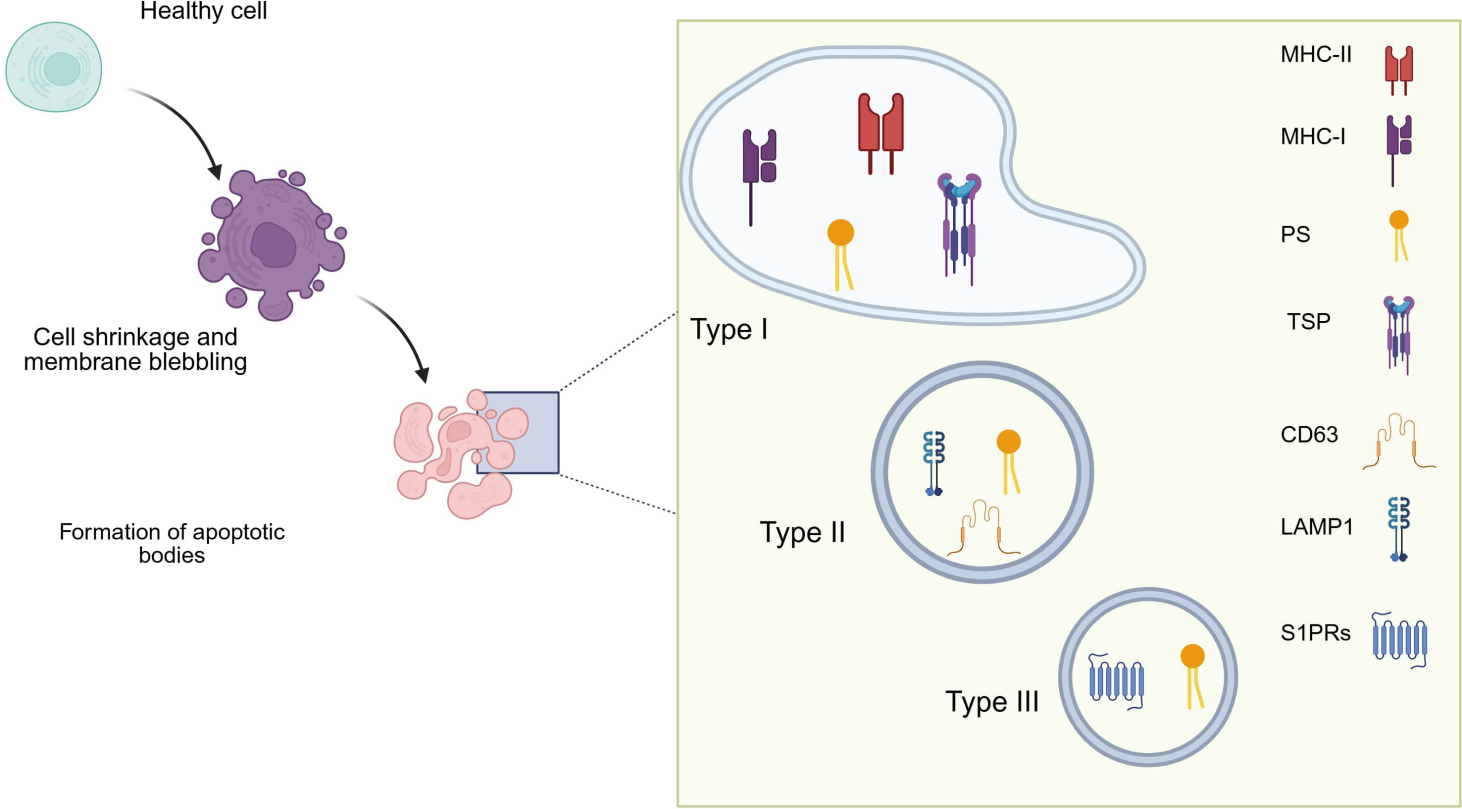
Different classes of ApoEVs contains different macromolecules.

ApoEVs contain nuclei debris, and structurally recognizable organelles and other cellular components such as proteins, RNA, and DNA, as shown by microscopy. Based on the cell type and apoptotic triggers, their nuclear and mitochondrial content varies and can be used to distinguish them, **Figure 1**, **Table 2**.

**Table 2:** Novel classification system for apoptotic extracellular vesicles.

Class	Type-I	Type-II	Type-III
Size Range	1-5 µm	200-1000 nm	50-200 nm
Primary Cargo	Nuclear fragments, organelles	Cytoplasmic proteins, limited nuclear material	miRNAs, specific proteins
Surface Markers	PS, MHC I/II, TSP	PS, CD63, LAMP1	PS, S1PR1, S1PR3, S1PR4
Functional Properties	Immunomodulatory, high DNA content	Pro-inflammatory, moderate DNA content	Signaling functions, low DNA
Typical Sources	Epithelial cells, fibroblasts	Immune cells, endothelial cells	Various cell types under specific stressors

The composition of ApoEVs further differs from that of other subtypes of EVs, including differences in surface markers expression. Besides the Major Histocompatibility Complexes I and II (MHC I, II), which are present on ApoEVs as well as other types of extracellular vesicles, ApoEVs are specifically enriched in complement proteins, and thrombospondin (TSP). Additionally, phosphatidylserine (PS) externalization onto ApoEV surfaces is a hallmark feature that serves as a phagocytic cell recognition signal (24).

Therefore, ApoEVs functional characteristics, cellular origin, and cargo composition, a comprehensive classification scheme is reported in **Table 2**. This framework provides a basis for understanding the heterogeneity of ApoEVs and their potential diagnostic applications. Type II and Type III ApoEVs may reflect inflammatory processes and dysregulated cell signaling in neurological disorders, whereas Type I ApoEVs, characterized by a high DNA content, may be particularly valuable for detecting genetic alterations in cancer.

## 3 Molecular cargo of ApoEVs: diagnostic biomarker potential

### 3.1 Protein biomarkers in ApoEVs

The complex cell events that take place during apoptosis induce a characteristic alteration of the cellular proteome that is faithfully reflected in the cargo of ApoEVs (1, 5). These vesicles contain structural proteins which mirror the cell state and anatomical site at the time of death, intracellular enzymes that help define the parental original cell type (5), and key regulators of the apoptotic signaling pathway (1).

Comparative proteomic analyses demonstrate that ApoEVs and extracellular vesicles released by viable cells (EVs) possess distinct surface-marker repertoires. While CEVs are enriched in CD9, Programmed Cell Death 6-interacting protein (PDCD6IP or ALIX), Ras-related protein (RAB7), and Sphingosine-1-phosphate receptors 2 (S1PR2), ApoEVs display higher levels of CD63, Lysosomal-associated membrane protein-1 (LAMP1), Heat shock protein70 (HSP70), and Sphingosine-1-phosphate receptors (S1PR1, S1PR3, S1PR4) (25).

These differences have important diagnostic implications. For instance, membrane proteins uniquely abundant on ApoEVs can serve as indicators of tissue injury from specific cell types, facilitating the monitoring of organ- or cell-targeted pathologies (1, 5). For example, vimentin, which is highly expressed on apoptotic T cells, has been detected at elevated levels in the serum of patients with rheumatoid arthritis, myocarditis, and systemic lupus erythematosus (SLE) probably deriving from T cell ApoEVs (26).

In one illustrative example, shotgun proteomics of ApoEVs derived from biliary epithelial cells identified eleven proteins, including Annexin A6, heat shock protein 6 (HSP6), and LDL receptor- related proteins, are specific to biliary epithelial cell ApoEVs and not found in EVs shed by healthy control cells. A possible role in autoimmune response is suggested by the interactions of these proteins with immunological pathways including NF-kB, ERK, and Notch signaling (27).

It is worth noting that ApoEV proteomic profiles vary with the cell type, the nature of the pro-apoptotic stimuli, and the surrounding pathological milieu. This heterogeneity represents a challenge for biomarker standardization but also offers the opportunity to develop highly specific and personalized diagnostic assays provided that candidate markers are validated across different patient cohorts.

### 3.2 Nucleic acid biomarkers in ApoEVs

New therapeutic avenues have been opened by the finding that ApoEVs carry DNA and RNA (1). Key genetic changes characteristic of diseases such as cancer chromosomal rearrangements, deletions, or mutations can be detected in DNA isolated from ApoEVs (3, 28).

ApoEV-derived DNA offers several advantages as biomarker source. Firstly, it is very stable under a range of storage conditions, making it suitable for clinical workflows with delayed sample processing. making it suitable for clinical workflows with delayed sample processing. Secondly, it enables non-invasive way of detection of tumor specific mutations and can support minimal residual disease monitoring, cancer diagnosis, and assessment of treatment response (3).

The miRNA and mRNA cargo profile of ApoEVs reveal insight into the gene expression regulation of apoptosis and has been investigated as a biomarker source across a number of disorders (1, 29). For instance, in mouse models, endothelial cell-derived ApoEVs enriched in miR-126 have been shown to slow the formation of atherosclerotic plaque by recruiting Sca-1+ progenitor stem cells and preserving endothelial integrity (30).

The fragmentation pattern of nucleic acids occurring during the apoptotic process and carried by ApoEVs may be an important biomarker feature. Indeed, the well-known nucleosome-sized DNA fragments with a visible peak at around 150–200 bp have been demonstrated to be present also in plasma ApoEVs (31). Numerous neurological disorders, such as multiple sclerosis, ischemic stroke, and Parkinson’s disease, exhibit this fragmentation pattern, reflecting a shared mechanism of DNA packaging in ApoEVs which may serve as a monitorable biomarker. Additionally, as reported in **Table 3**, heterogeneity exists among the different types of nucleic acids within ApoEVApoEVs cargo, increasing their applications in diagnostics of diseases. Although each class offers distinct advantages, there are also certain technological issues that need to be resolved prior to their use in therapy applications.

**Table 3:** Comparison of nucleic acid biomarkers in ApoEVs.

Nucleic acid type	Characteristics	Diagnostic applications	Advantages	Limitations
DNA	Fragmented (50-200 bp), nucleosomal pattern.	Cancer mutations, chromosomal alterations.	Stable, reflects genetic changes.	Hinders large-region analysis.
mRNA	Partially degraded, key transcripts intact.	Gene expression changes in disease.	Reflects cellular state at death.	Inherently less stable than DNA.
miRNA	Well-preserved, miRNA-enriched.	Disease-specific miRNA signatures.	Highly stable, regulatory functions.	Requires sensitive detection methods.
Other ncRNAs	Variable preservation.	Emerging applications.	Diverse regulatory functions.	Insufficient clinical validation.

Recent developments suggest new avenues for the generation of biomarkers by exploiting the natural DNA packaging mechanisms in ApoEVs through endogenous proteases. In apoptotic cells and, by extension, in ApoEVs, proteases like matrix metalloproteinase-2 (MMP-2) and caspase-3 (Casp-3) regulate DNA assembly, and this process is reflected in the DNA content of ApoEVs. By monitoring these enzymes as endogenous stimuli can leverage this natural mechanism to develop biomarkers (32). These strategies can find application in metastatic monitoring, response to treatment monitoring, and disease diagnosis. For example, one promising method for monitoring metastases is the controlled assembly of DNA in ApoEVs through protease activity (e.g., MMP-2 and caspase-3). ApoEVs can produce dynamic biomarkers that represent tumor aggressiveness, metastatic spread, and treatment response by using these enzymes as endogenous stimuli. With liquid biopsies, this method allows for non-invasive, real-time tracking of disease progression, which may enhance early detection and individualized treatment plans.

This analogy demonstrates ApoEVs’ heterogeneity in nucleic acid cargo and their applications in diagnostics of diseases. Although there are distinct benefits to each class, there are also certain technological issues that need to be resolved prior to their use in therapy applications.

## 4 Detection and isolation methods: critical comparison

### 4.1 Current isolation techniques: strengths and limitations

Given ApoEV heterogeneity in size, density, and composition, their isolation poses significant technical challenges. Some of the recent isolation techniques include differential centrifugation, density gradient centrifugation, filtration, immune-affinity capture, and microfluidic platforms. The purity, yield, and integrity of isolated ApoEVs depend on the strengths and limitations of each isolation technique as it demonstrated in **Table 4**.

**Table 4:** Critical comparison of ApoEV isolation techniques.

Method	Principle	Advantages	Limitations	Purity	Yield	Clinical applicability
Differential Centrifugation	Sequential centrifugation based on size/density.	Simple, widely accessible.	Time-consuming, aggregation risk, contamination.	Low-Medium	Medium	Limited by equipment requirements.
Density Gradient Centrifugation	Density-based separation in a gradient medium.	Higher purity, separates ApoEVs from other EVs.	Labor-intensive, low throughput, density overlap.	High	Low	Time-consuming and complex.
Filtration	Size-based separation using membranes.	Rapid, scalable.	Membrane clogging, vesicle deformation.	Medium	Medium	Good for preliminary enrichment.
Size Exclusion Chromatography (SEC)	Size-based separation using porous beads.	High purity, maintains vesicle integrity.	Poor size-resolution, requires dilution.	High	Medium	Ideal for research, not clinically scalable.
Immunoaffinity Capture	Antibody-based capture of specific surface markers.	High specificity, captures subpopulations.	Costly, marker-specific, potentially disruptive.	Very High	Low	Promising (standardization needed).
Microfluidic Devices	Size/immuno-affinity-based, automated on a chip.	Minimal sample volume, high precision.	Limited throughput, specialized equipment.	High	Medium	High potential (needs validation).
PANX1 Inhibition + Apoptopodia Isolation	Yield enhanced by apoptosis induction.	High yield, high homogeneity.	Non-standardized, potentially artifactual.	Medium-High	High	Experimental, requires validation.

A variety of methods, each with distinct advantages and limitations, are used in the isolation of ApoEVs. Although differential centrifugation is easy to use and accessible, its clinical utility is limited by the low-to-medium purity it produces due to contamination risks (33). Density gradient centrifugation improves purity, but it is time-consuming and has a low throughput (34). Although size exclusion chromatography (SEC) offers high purity and preserved vesicle integrity by separating vesicles based on size, it has low resolution for particles of similar sizes and necessitates large sample volumes (35). Filtration devices such as Exodiscs (36) offer a quick and scalable method, but can cause vesicle deformation and clogging (37). Immunoaffinity capture is expensive and marker-dependent, nonetheless it offers high specificity for subpopulations (38). In the case of microfluidic devices, they lack throughput and need validation, but microfluidic devices allow automation and minimal sample use (39). Notably, apoptopodia isolation in conjunction with PANX1 inhibition shows promise as an experimental technique, providing high yield and homogeneity but requiring standardization (40, 41). While microfluidics and PANX1 inhibition demonstrate innovation in clinical translation, SEC and immunoaffinity stand out for purity; however, scalability and reproducibility continue to be major obstacles. Depending on the sample type, downstream analysis, and the working conditions, researchers should select the appropriate separation technique (42).

No one technique is best for every application, as illustrated by this thorough review. The research or clinical query, needed purity and yield, and resources available must all be taken into consideration when selecting a method of isolation. One might find a combination of methods to be the best compromise of convenience, yield, and purity for clinical use.

### 4.2 Detection and characterization methods: sensitivity and specificity analysis

ApoEVs must be accurately detected and characterized in order to be used as biomarkers. Currently used techniques include electron microscopy, molecular assays (such as qPCR), flow cytometry, imaging flow cytometry, and nanoparticle tracking analysis. Although the sensitivity, specificity, and technical requirements of each approach vary, they all provide unique insights into ApoEV characteristics.

Among the different techniques, flow cytometry is the most frequently used for ApoEV analysis due to its ability to simultaneously analyze multiple markers through multiparametric analysis (43). The detection of small ApoEVs is diffiicult as well as the separation from background noise using standard flow cytometry. Standard flow cytometry typically struggles to detect particles below 200–300 nm due to limitations in light scattering sensitivity. However, dedicated small-particle or high-resolution flow cytometers have been developed, enabling detection of particles as small as 100 nm, including small extracellular vesicles like ApoEVs. Moreover, integrating flow cytometry and microscopy, imaging flow cytometry addresses some of these disadvantages and enables the visual confirmation of events (44).

While standard nanoparticle tracking analysis (NTA) can be helpful for determining particle size and concentration, it does not offer information on marker expression (45). Additionally, electron microscopy (EM) provides high-resolution imaging of ApoEV morphology, its low throughput and limited suitability for routine clinical application constrains its practical use (46). Artificial Intelligence (AI)-assisted video microscopy, which use deep convolutional neural networks to automatically identify apoptotic bodies and cells in label-free time-lapse microscopy, is one example of a recent development in detection techniques (47).This method provides high-throughput analysis with little sample preparation, although it needs to be validated in various cell types and disease scenarios to pass the checklist of “EV-TRACK: transparent reporting and centralizing knowledge in EVs research” (48). pros and cons of each detection method in the case of sensitivity and specificity is demonstrated in **Table 5**.

**Table 5:** Sensitivity and specificity analysis of ApoEV detection methods.

Method	Size detection limit	Markers analyzed	Quantitative capability	Single vesicle analysis	Throughput	Technical complexity	Cost
Conventional Flow Cytometry	~300-500 nm	Surface proteins	High	Limited	High	Medium	Medium
Imaging Flow Cytometry	~200 nm	Surface and internal markers	High	Yes	Medium	High	High
Nanoparticle Tracking Analysis	~50 nm	Limited	High	No	Medium	Medium	Medium
Electron Microscopy	~1 nm	Limited	Low	Yes	Low	Very High	High
Mass Spectrometry	N/A	Protein composition	High	No	Medium	Very High	Very High
Digital PCR	N/A	Nucleic acids	Very High	No	Medium	High	High
AI-Assisted Microscopy	Variable	Morphological features	Medium	Yes	High	Medium	Medium

### 4.3 Emerging technologies and standardization proposals

Several new technologies are being promising for increased sensitivity, specificity, and clinical value in the fast-moving field of ApoEVs detection and isolation. These include:

#### 4.3.1 Single-vesicle sequencing

This platform permits to examine the nucleic acid content of single vesicle, generating information on ApoEVs heterogeneity and enabling detection of rare variants (49–51).

#### 4.3.2 Nanoparticle-based sensors

Sensors show a strong specificity for the detection of certain EVs indicators, possibly enabling point-of-care diagnostics. Nanoparticle with various electrochemical and fluorescent properties for detecting EVs. Nanoparticle such as tethered cationic lipoplex nanoparticles (tCLN) showed promising results to distinguish between non-tumor exosomes versus tumoral exosomes through their miRNA contents (52, 53).

#### 4.3.3 Microfluidic isolation platforms

Platforms combine numerous processes of isolation and detection into one device, with reduced processing time and volume demands. In this method both chemical and physical properties of the EVs are used to purify them. Moreover, microfluidics can be integrated with other techniques such as immuno-affinity and nanoparticle to study the Evs (54).

#### 4.3.4 Raman spectroscopy

The label-free technology does not involve any particular markers to reveal the metabolic content of ApoEVs. In this case platform that analyzes individual EVs separated by size-exclusion chromatography (SEC) by integrating machine learning and single-vesicle surface-enhanced Raman spectroscopy (SERS). The difficulties caused by EV population averaging were successfully overcome by SERS fingerprinting of individual vesicles, which made it possible to examine the differences in biomolecular composition between vesicles of comparable and/or varying sizes (55).

In spite of these developments, a major drawback in ApoEV research and clinical practice is the absence of standardization.

According to the Minimal Information for Studies of Extracellular Vesicles 2018 (MISEV2018) standards, to ensure consistency and reproducibility in ApoEV research, standardized procedures for sample collection, processing, and characterization are essential. Standardized preservative-containing tubes must be used for sample collection and processing, and a two-hour processing window or prompt storage at -80 °C must be strictly followed. It is important to fully document all pre-analytical factors, such as handling conditions and collection time. Equipment and methodology, such as density gradient separation, differential centrifugation (300g and 2000g steps), and, if appropriate, immunoaffinity purification for target-specific enrichment, must be thoroughly described in the isolation protocol. Size distribution profiling and the detection of a minimum of three markers, including phosphatidylserine (PS), a cell-type marker, and an ApoEV-specific protein, in addition to negative controls to confirm sample purity, are necessary for characterization. Lastly, in order to guarantee reproducibility and cross-study comparability, reporting requirements need to incorporate thorough methodological documentation, uniform numeric units, and strict quality control methods.

This uniform method would speed up the clinical validation of ApoEV-based biomarkers and make it easier to compare research. With particular attention on ApoEVs, it complements and expands upon the MISEV2018 standards (13).

## 5 ApoEVs in cancer diagnostics: critical analysis and comparative evaluation

### 5.1 Current state of ApoEV-based cancer biomarkers

ApoEVs have very significant clinical potential in cancer diagnostics. ApoEVs harboring tumor-specific biomarkers are released into the bloodstream by cancer cells going through apoptosis, either naturally or in response to treatment (28, 56). These biomarkers offer important insights about the nature of tumors, the effectiveness of treatments, and the course of the disease.

EVs-derived biomarkers have shown diagnostic potential across various types of cancer. For instance, It can be possible that EpCAM-positive ApoEVs indicate colorectal cancer (57) CD138-positive microparticles assist in diagnosing multiple myeloma (58) KRAS-mutated EVs-DNA supports early detection of pancreatic cancer (59) miRNA signatures in plasma vesicles help monitor Hodgkin lymphoma treatment response (60) TrpC5-EVs are linked to chemoresistance in metastatic breast cancer (61) and urinary EVs included ApoEVs serve as protein biomarkers for prostate cancer diagnosis and prognosis (62, 63).

Nevertheless, a careful review of the literature identifies a number of shortcomings and inconsistencies in the state of the field. Results may be skewed by the fact that many studies do not differentiate ApoEVs from other EV subtypes. Furthermore, there is significant variation in the specificity of suggested biomarkers among research, with some exhibiting overlap between cancer and non-cancer illnesses. It sounds some of the biomarkers that mentioned as cancer biomarkers can be presented in healthy ApoEVs and EVs too. Additionally, majority of papers have not drawn a line between EVs and ApoEVs.

### 5.2 Comparative analysis: ApoEVs vs. established cancer biomarkers

We must contrast ApoEVs with well-established methods like circulating tumor cells (CTCs), cell-free DNA (cfDNA), and traditional protein biomarkers in order to assess their potential as cancer biomarkers.

ApoEVs differ from other liquid biopsy components in both their advantages and disadvantages. ApoEVs come from apoptotic cancer cells and are not as stable CTCs, which come from intact cancer cells but are uncommon in very early-stage disease and technically difficult to isolate. CTCs need a large number of samples (64). Moreover, in comparison to the full payload (DNA, RNA, proteins) of ApoEVs, this durability stands in contrast to the fragility of cfDNA and the moderate half-life of cfDNA, which, although important, offers limited molecular information (DNA-only) (65).

ApoEVs exhibit greater promise for early identification than both conventional protein markers (typically raised only in advanced disease) and CTCs (sometimes undetected in early stages). However, because of its wider cellular origins, cfDNA may more accurately reflect clonal diversity than ApoEVs, which only partially capture tumor heterogeneity (66). Although ApoEV profiling is technically less resource-intensive than CTC isolation, which necessitates specialist platforms like CellSearch^®^, it is still more difficult than protein marker assays (67).

Although their multi-analyte cargo (such as tumor-specific RNAs + surface proteins) offers unique diagnostic relevance, ApoEVs clinically lag behind CTCs and cfDNA in validation (68). A brief comparison between the analysis of cancer biomarker was described in **Table 6**. This comparison draws attention to a number of possible benefits of ApoEVs, such as their early detection potential, stability, and rich information content. In contrast to well-established biomarkers, ApoEVs also have limitations in terms of clinical validation and technical complexity.

**Table 6:** Comparative analysis of cancer biomarker approaches.

Parameter	ApoEVs	CTCs	cfDNA	Protein
Origin	Apoptotic cells	Intact tumor cells	Various (apoptosis, necrosis)	Various
Stability	High	Low	Medium	Variable
Information Content	Inclusive (DNA, RNA, protein)	Very high (whole cells)	DNA only	Limited
Early Detection Potential	High	Limited	Medium-High	Variable
Reflection of Tumor Heterogeneity	Partial	Limited	Good	Limited
Technical Complexity	Medium-High	Very High	Medium	Low
Level of Clinical Validation	Limited	Established	Growing	Established
Approximate Cost	Medium-High	Very High	High	Low-Medium

### 5.3 Conflicting findings and controversies

The emerging field of ApoEVs as a potential source of cancer biomarkers is highly promising but not yet fully established. Indeed, several disputes and contradictory results still persist on the following aspects. The area of ApoEVs as a possible source of cancer biomarkers is still in its infancy. The origin of circulating DNA is a major topic of controversy. While some studies contend that ApoEVs are primarily responsible for tumor-derived circulating DNA, others counter that the majority of circulating tumor DNA is non-vesicular, possibly as a result of variations in isolation methods and the cancer types studied (69). Furthermore, there is still debate on the specificity of ApoEVs markers since while some studies emphasize their great sensitivity for detecting cancer, other studies show substantial overlap with inflammatory and other disorders, casting doubt on the accuracy of the diagnosis (1).Uncertainty surrounds the association between ApoEVs and treatment response as well (70). The necessity for bigger, rigorously standardized trials is further highlighted by the fact that standardization issues, such as inconsistent isolation and characterization techniques, make it difficult to draw firm conclusions regarding the clinical utility of ApoEVs in cancer detection.

Altogether, these open questions emphasize that future research should address such inconsistencies by ensuring strict clinical validation, thorough characterization of ApoEVs subtypes, and improved separation methods for their isolation and analysis.

### 5.4 Case study: ApoEVs in pancreatic cancer diagnostics and treatment monitoring

As a result of its poor prognosis and the critical need for early detection, pancreatic cancer represents a particularly compelling application for ApoEVs-based biomarkers. *In vitro* studies have shown that pancreatic cancer cells treated with gemcitabine release increased levels of ApoEVs, offering a window for therapy monitoring. Moreover, ApoEVs-derived DNA can reveal KRAS mutations, present in over 90% of pancreatic ductal adenocarcinomas, thereby enabling early detection and monitoring of minimal residual disease would be feasible. However, it is still diffiicult to distinguish tumor-derived ApoEVs and normal cell-derived ApoEVs undergoing apoptosis within the inflammatory milieu of pancreatic cancer. The detection of pancreatic cancer-derived ApoEVs has been improved by recent advances in microfluidic technologies. For example, impedance cytometry measurement of ApoEVs can assess drug sensitivity of pancreatic tumor cell lines in a label-free manner, illustrating a promising approach for real-time monitoring therapy response that could be translated to the clinics (71).

While these developments look promising, some problems need to be solved before ApoEV- based strategies can be implemented for pancreatic cancer. These include increasing specificity to discriminate between benign and malignant conditions, improving sensitivity to detect early stages disease, and establishing standardized protocols for sample collection and handling.

## 6 ApoEVs in neurodegenerative diseases: from biomarkers to mechanistic insights

### 6.1 ApoEVs as biomarkers for neurodegenerative disorders

Since the blood-brain barrier and the Accessibility to the involved tissues are restricted, neurodegenerative diseases present unique problems in diagnosis. ApoEVs provide a potential method of non-invasively tracking disease progression and neuronal damage. The potential of ApoEVs as biomarkers for the disease conditions multiple sclerosis (MS), Parkinson’s disease (PD), and Alzheimer’s disease (AD) has been under investigation by several investigations.

Pathogenic proteins in neutrally derived blood exosomes and EVs in Alzheimer’s disease have been shown to identify preclinical AD (72). Amyloid-42 (A-42), phosphorylated tau (P-T181-tau, P-S396-tau), and dysfunctionally phosphorylated type 1 insulin receptor substrate (IRS-1) are only a few of them (72, 73). A less invasive option to cerebrospinal fluid testing is the identification of these markers in blood-derived ApoEVs.

Similarly, plasma levels of neuron-and glia-derived ApoEVs correlate with infarct size and patient functional outcome in ischemic stroke, suggesting that ApoEVs cane serve as apoptosis markers *in vivo*, with prognostic value in neurological disease (74).

Close inspection does identify a number of flaws in the present study. Numerous obstacles restrict the potential of neural-derived EVs, such as ApoEVs, as biomarkers for neurodegenerative disorders. Because different isolation strategies produce different EVs populations, methodological variation makes cross-study comparisons more difficult (75, 76). Furthermore, there are still problems with specificity because the neuronal markers that are now employed to identify brain-derived ApoEVs in peripheral blood might not be selective enough, requiring additional research. Their potential for staging neurodegenerative disorders is hampered by the lack of evidence linking EVs levels with disease development and the incomplete understanding of the temporal dynamics of EVs release (77). Additionally, interpretation can be complicated by confounding factors that can change ApoEVs profiles independently of neurodegeneration, such as comorbidities (e.g., cardiovascular disorders) frequent in neurodegenerative patients (78).

### 6.2 Comparative analysis: ApoEVs vs. established neurological biomarkers

We contrast ApoEVs with well-established biomarkers including cerebrospinal fluid (CSF) analysis, neuroimaging, and traditional blood biomarkers in order to assess their potential in the diagnosis of neurodegenerative diseases. A comparison of different methods are applied were summarized in the **Table 7**.

**Table 7:** Comparison of neurodegenerative disease biomarker approaches.

Parameter	ApoEVs	CSF Analysis	Neuro-imaging	Typical blood biomarkers
Invasiveness	Minimally invasive (blood)	Invasive (lumbar puncture)	Non-invasive but expensive	Minimally invasive (blood)
Specificity for Neurodegeneration	Medium-High	Very High	High	Low-Medium
Sensitivity for Early Detection	Potentially high	High	Variable (depends on technique)	Generally low
Diagnostic Specificity	Emerging evidence	Good	Good for structural changes	Limited
Reflection of Disease Mechanisms	Direct (cellular contents)	Direct (brain-derived)	Indirect (structural/functional)	Variable (often indirect)
Technical Complexity	High	Medium	High	Low
Current Level of Clinical Validation	Limited	Well-Established	Well-Established	Variable
Approximate Cost	Medium-High	Medium	Very High	Low

ApoEVs offer a viable, but unproven, substitute for recognized indicators of neurodegenerative diseases. EVs detection provides a less intrusive blood-based method than costly neuroimaging approaches or invasive cerebrospinal fluid (CSF) analysis, which requires lumbar puncture (79). EVs exhibit medium-high specificity with the distinct benefit of having direct cellular contents that may more accurately reflect disease mechanisms, whereas CSF biomarkers show very high specificity for neurodegeneration and neuroimaging offers excellent structural assessment (77). EVs may provide high sensitivity for early identification, potentially outperforming traditional blood biomarkers, which often exhibit low-medium sensitivity, according to preliminary data (80). In contrast to the known differential diagnostic capabilities of CSF analysis and sophisticated neuroimaging, their capacity to differentiate between various neurodegenerative illnesses is still being studied (81). In contrast to more straightforward, low-cost blood tests, EVs analysis is still technically complex and medium-to-highly expensive, but it provides more direct pathological information than traditional serum indicators (82). EVs-based diagnoses need more standardization and extensive validation studies prior to routine clinical application, whereas CSF analysis and neuroimaging have broad clinical validation (83).

According to this comparison, ApoEVs may be able to supplement current strategies by providing a minimally invasive technique that has the potential to be highly sensitive for early detection and direct reflection of disease causes. To prove their specificity and capacity to differentiate between various neurodegenerative diseases, more research is necessary. As majority research does not differentiate between EVs and ApoEVs.

### 6.3 Mechanistic insights: ApoEVs in neurodegeneration pathophysiology

More recent studies indicate that ApoEVs may be involved in the pathogenesis of neurodegenerative disorders by enabling the disease transmission by transferring toxic proteins from one cell to another. Indeed, Tau protein-carrying ApoEVs can drive tau pathology transmission throughout the brain in Alzheimer’s disease (84, 85). Similarly, ApoEVs carrying α-synuclein can drive Lewy body pathology transmission in Parkinson’s disease. These processes indicate that ApoEVs have active roles in disease etiology rather than being a mere consequence of neurodegeneration (86–88).

ApoEVs’ immunomodulatory function also promotes neuroinflammation, a shared aspect of neurodegenerative conditions. Based on the cargo and target cells, ApoEVs can stimulate or inhibit inflammatory responses that can influence the severity and progression of the disease (89–92).

These mechanistic findings imply that, beside play a role as biomarkers, ApoEVs have a potential in neurodegenerative illnesses as therapeutic targets. Potential approaches could be directed to modulate their effect by modifying ApoEV synthesis, cargo loading, or absorption.

### 6.4 Case study: ApoEVs in Alzheimer’s disease diagnosis and monitoring

Due to the urgent need for early biomarkers and the diffiiculties associated with obtaining brain tissue, Alzheimer’s disease represents a compelling study case for the application of ApoEV. Blood- based ApoEVs secreted by neurons can be identified in preclinical phases of disease (92).

According to Fiandaca et al., a pathogenic protein profile of blood exosomes derived from neutrally is able to consistently differentiate between AD patients and frontotemporal dementia patients and healthy controls (72). P-T181-tau, P-S396-tau, and Aβ1-42, recognized markers of AD pathology, were included in the biomarker panel.

In addition, Kapogiannis et al. demonstrated that exosomes from neurons that bore dysfunctionally phosphorylated type 1 insulin receptor substrate (IRS-1) were able to forecast the development of AD up to ten years before its onset (73). This evidence implies that ApoEVs-based monitoring has the potential to interventions prior irreversible effects of neurodegeneration.

These promising findings highlight the significant potential of ApoEVs, which could be fully realized through ongoing improvements in isolation specificity, standardized protocols, and longitudinal validation to track biomarker dynamics over time. Integrating ApoEV-based diagnostics with established modalities such as neuroimaging and genetic risk assessment may further increase diagnostic precision and accelerate their adoption in clinical settings.

## 7 Challenges in standardization and clinical translation

### 7.1 Technical and biological variability

Despite the considerable promise of ApoEV-based biomarkers, several technical and biological sources of variability still need to vibe addressed to ensure reliable clinical translation. Pre-analytical factors such as sample handling, blood collection tubes, processing delays, freeze-thaw cycles and storage conditions can all affects ApoEVs integrity and recovery, while heterogeneity of isolation methods may yield preparations with different purity, yield, and representation of particle subpopulations. Moreover, biological variables-, including circadian rhythm, exercise, diet, and the intrinsic heterogeneity of diseases like cancer or neurological disorders-, further complicate consistent biomarker measurement. ApoEV pro- files can be all affected by disease stages, subtypes, and patient factors. To overcome these challenges, a comprehensive standardization is essential. This include, the adoption of rigorous standard operating procedures for sample collection and processing, the development of reference materials for method validation, the integration of robust; quality control procedures through- out the analytical process, and the implementation of normalization strategies to account for biological variability (93).

### 7.2 Regulatory considerations and validation requirements

Several factors are being taken into account in the process of ApoEVs-based diagnostics regulation:

According to the FDA’s Bioanalytical Method Validation Guidance (2018) and MISEV2018 guidelines, development of ApoEVs as clinical diagnostics necessitates careful navigation of regulatory benchmarks, starting with analytical validation to demonstrate method sensitivity, specificity, accuracy, and precision in ApoEV isolation and characterization (13). Following models such as the EMA’s Guideline on Clinical Evaluation of Diagnostics (2017) and neuronal EV studies in Alzheimer’s disease (72), clinical validation must then demonstrate diagnostic accuracy and predictive value through multicenter trials. With guidelines from the NIH Extracellular RNA Communication Consortium for EV characterization, strict quality control procedures including pre-analytical to analytical stages are essential (94).The area also needs universal reference materials, which are being addressed by programs like NIST’s RM 8640 and EV-TRACK (48). Moreover, regulatory systems such as the Food and Drug Administration(FDA) and European Medicines Agency (EMA) require analytical validity through method validation studies, clinical validity through prospective trials, and demonstrated clinical utility through outcome studies (95). As demonstrated by consortia like ISEV’s Rigorous Science initiative (93), which together seek to standardize and validate ApoEV-based diagnostic approaches, successful translation will ultimately depend on cooperative efforts across academia, industry, and regulatory bodies.

## 8 Future directions and novel applications

Looking ahead, artificial intelligence and machine learning hold great promise for addressing some of the main issues relating to ApoEV research and clinical application. By handling complex and multi-dimensional data, AI could improve detection accuracy, help classify diverse ApoEV subtypes, and integrate multi-omics information into more precise diagnostic models. Recent studies, for example, have explored the use of deep learning for automated detection of apoptotic bodies in label-free time-lapse microscopy, highlighting the potential for high-throughput analysis with minimal sample preparation. To translate these advances into practice, future work should focus on building standardized training datasets, developing explainable AI tools, and validating models across different clinical contexts. While still in its early stages, AI may become a valuable ally in bringing ApoEV-based diagnostics closer to the clinic (47).

In parallel, emerging biosensor technologies offer exciting opportunities to enhance ApoEV detection by making it more sensitive, precise, and accessible. Innovations such as surface plasmon resonance (SPR) sensors, which use label-free optical detection with specific antibodies or aptamers, and electrochemical biosensors, which measure electrical signals generated by ApoEV interactions, are advancing rapidly. Nanopore-based sensors can analyze individual vesicles as they pass through nanoscale pores, providing insights into their size, charge, and potentially even their molecular content. Another promising example is the use of tapered microfibers coated with graphene oxide for high-sensitivity detection of microscopic particles, which could be adapted for ApoEV applications. By enabling point-of-care use, these biosensor approaches could help reduce the costs and technical demands of current ApoEV analysis techniques. However, further research is needed to ensure their validation, reproducibility, and specificity when used with complex biological samples (96).

Additionally, ApoEVs have great potential as adaptable therapeutic agents in addition to their diagnostic uses. Because of their inherent biocompatibility and targeting ability, engineered ApoEVs can serve as extremely efficient drug delivery vehicles, delivering therapeutic payloads to target tissues while reducing off-target effects. Through carefully crafted formulations, their immunomodulatory qualities allow for dual uses, either boosting anticancer immunity or inhibiting aberrant immune responses in autoimmune illnesses. Stem cell-derived ApoEVs have shown impressive tissue repair capabilities in regenerative medicine; studies have shown that they are effective in musculoskeletal, cardiac, and brain regeneration (97). ApoEVs are also positioned as novel vaccination platforms due to their intrinsic immunogenicity, especially when they are designed to exhibit pathogenic fragments or tumor-specific antigens. As demonstrated by miR-21-5p-enriched ApoEVs from M2 macrophages demonstrating focused efficacy in osteoarthritis treatment by macrophage polarization regulation, recent research emphasizes their therapeutic precision (98). The requirement for standardized GMP-compliant production procedures, the improvement of drug-loading efficiency, the creation of tissue-specific targeting systems, and the negotiation of intricate regulatory processes for biological therapies are some of the difficulties that clinical translation must overcome. To fully utilize ApoEV-based treatments, interdisciplinary cooperation will be necessary to overcome these obstacles.

Finally, the personalized nature of ApoEVs makes them a promising tool for individualized medicine. Patient-specific ApoEV profiles could help guide treatment choices, indicating which therapies are most likely to succeed. Serial monitoring could provide real-time information on treatment response and disease progression, while ApoEV patterns could help identify patients at higher risk of complications or faster disease advancement. In oncology, ApoEV-derived DNA can be screened for tumor-specific mutations to guide targeted therapies and track resistance. Similarly, ApoEV protein signatures in neurodegenerative diseases could help pinpoint distinct disease mechanisms that may be addressed with personalized interventions. To fully realize this potential, new cost-effective and high-throughput methods of analysis, integration with other clinical and molecular data, and the development of decision-support tools will be key, alongside robust prospective clinical trials to demonstrate real-world benefits (99).

## 9 Conclusion

ApoEVs, which represent an emerging though underexplored subpopulation of extracellular vesicles, deserve to be examined in more detail regarding their potential biomedical and diagnostic use. Given that, going forward, ApoEVs are likely to carry the signature of the cells from which they originate, especially in cancers or other neurodegenerative diseases where there are a few but reliable biomarkers. The recent development of technologies necessary for the isolation, molecular characterization, and imaging of ApoEVs will pave the way for their application in translational medicine. However, much needs to be settled yet, like the establishment of SOPs (standard operating procedures), analytical sensitivity and specificity or massive validation studies in large cohorts.

**Figure 2:**
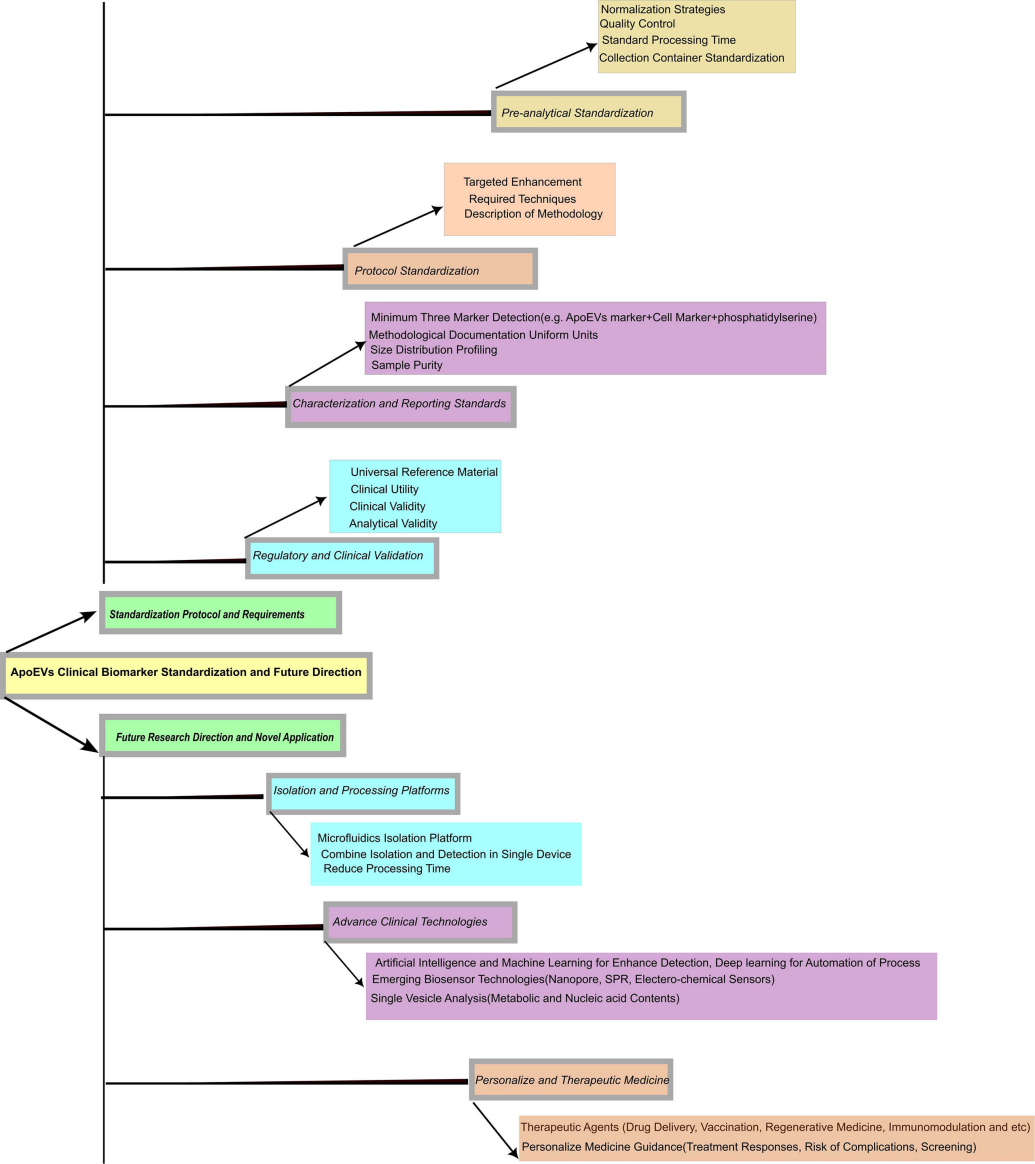
Roadmap from standardized diagnostic protocol to next-generation research and clinical translation.

The expected introduction of novel technologies such as AI-aided analysis, next-generation biosensors, and personalized diagnostic platforms will probably facilitate clinical applications of ApoEVs-based diagnostic and therapeutic devices. Basic sciences, clinical development, and novel technologies will be necessary to realize this significant clinical potential of the ApoEVs. With further profound studies, ApoEVs will come to the forefront of clinical applications. At the end, to enhance the clinical translation of ApoEVs we have provided a roadmap **Figure 2**. This roadmap demonstrates how the application of ApoEVs from pre-analytical steps to multi-parametric characterization. Additionally, it clearly outlines the trajectory toward Next-Generation Research Directions (AI-Assisted Analysis, Biosensors, etc.).
